# Nuclear formation induced by DNA-conjugated beads in living fertilised mouse egg

**DOI:** 10.1038/s41598-019-44941-6

**Published:** 2019-06-11

**Authors:** Yuka Suzuki, Şükriye Bilir, Yu Hatano, Tatsuhito Fukuda, Daisuke Mashiko, Shouhei Kobayashi, Yasushi Hiraoka, Tokuko Haraguchi, Kazuo Yamagata

**Affiliations:** 1 Faculty of Biology-Oriented Science and Technology (BOST), Kindai University, Nishimitani 930, Kinokawa-shi, Wakayama 649-6493 Japan; 2 Graduate School of Frontier Biosciences, Osaka University, 1-3 Yamadaoka, Suita, 565-0871 Japan; 3 Advanced ICT Research Institute Kobe, National Institute of Information and Communications Technology, 588-2 Iwaoka, Iwaoka-cho, Nishi-ku, Kobe 651-2492 Japan

**Keywords:** Nuclear organization, Nuclear envelope

## Abstract

Reformation of a functional nucleus at the end of mitosis is crucial for normal cellular activity. Reconstitution approaches using artificial beads in frog egg extracts have clarified the molecules required for nuclear formation *in vitro*. However, the spatiotemporal regulation of these components, which is required for the formation of a functional nucleus in living embryos, remains unknown. Here we demonstrate that exogenous DNA introduced in the form of DNA-conjugated beads induces the assembly of an artificial nucleus in living mouse cleavage-stage embryos. Live-cell imaging and immunofluorescence studies revealed that core histones and regulator of chromosome condensation 1 (RCC1) assembled on the DNA, suggesting that nucleosomes were formed. Electron microscopy showed that double-membrane structures, partly extended from annulate lamellae, formed around the beads. Nuclear pore complex-like structures indistinguishable from those of native nuclei were also formed, suggesting that this membranous structure resembled the normal nuclear envelope (NE). However, the reconstituted NE had no nuclear import activity, probably because of the absence of Ras-related nuclear protein (Ran). Thus, DNA is necessary for NE reassembly in mouse embryos but is insufficient to form a functional nucleus. This approach provides a new tool to examine factors of interest and their spatiotemporal regulation in nuclear formation.

## Introduction

In eukaryotes, genomic DNA is compacted into nucleosomes and is housed in the nucleus in the form of chromatin. Nucleosomes are the basic repeating units of chromatin, in which 146 base pairs of DNA are wrapped twice around a histone octamer composed of two molecules of each of the core histones, H2A, H2B, H3 and H4^1,2^. The chromatin is enclosed by the nuclear envelope (NE) comprising inner and outer nuclear membranes. Many nuclear pores punctate the NE at points where the inner and outer membranes are merged. The nuclear pore is composed of a large protein structure called the nuclear pore complex (NPC); this acts as a gateway for traffic between the nucleus and the cytoplasm^3^. Molecules smaller than ~50-60 kDa, such as ions and small proteins, can diffuse through the NPC passively, while larger molecules are excluded^4,5^ or can be selectively and actively transported through the NPC dependent on their binding to nuclear transport receptors, the importin β superfamily proteins^6–8^. In metazoans, a nuclear lamina exists beneath the inner nuclear membrane in addition to these fundamental structures. This is a filamentous meshwork structure composed of type V intermediate proteins called lamins^9–11^. It is known that mutations in the gene encoding lamin A cause various inheritable diseases, such as Emery-Dreifuss muscular dystrophy, Charcot-Marie-Tooth disease and Hutchinson-Gilford progeria syndrome^12,13^. Thus, the normal organisation of the NE, including the nuclear lamina, is important for normal cellular functions in humans. The NE is a dynamic structure that undergoes repeated breakdown and reformation during cell division cycles. This dynamic reformation of the NE plays an important role in re-establishing the architecture of the functional nucleus^14^.

Several attempts have been made to elucidate the mechanisms and factors involved in NE reformation. One approach was to visualise the dynamic process of cell division using various imaging technologies including fluorescence live-cell imaging, electron microscopy and their combination^15,16^. Others have combined these techniques with other methods such as depleting or mutating the proteins of interest. Hence, fluorescence live-cell imaging has revealed the molecular mechanisms and the factors involved in reformation of the NE during mitosis. One such molecule is the barrier-to-autointegration factor (BAF), a chromatin-binding protein^17,18^. BAF accumulates at distinct regions of the telophase chromosomes, called ‘core regions’, at the beginning of telophase and plays a role in the assembly of LAP2, emerin, MAN1 (LEM) domain NE proteins such as emerin^16,19–21^. ELYS, one of the nucleoporins comprising the NPC, is involved in NE reformation at the non-core regions of the late telophase NE^22^. One of the endosomal sorting complexes required for transport (ESCRT) proteins, ESCRT-III, plays a role in NE reformation during telophase by sealing the gaps or pores in the reassembling membranes^15^.

Other attempts to determine the key factors required for NE reformation involved *in vitro* experiments using *Xenopus* egg extracts, in which molecules of interest were conjugated to beads (about 100 μm in diameter) and incubated in extracts from unfertilised eggs to visualise formation of nuclei around the beads. To date, this system has identified double-stranded DNA (dsDNA), Ras-related nuclear protein (Ran) and importin β as key factors^23–26^; however, in this system, the regulator of chromosome condensation 1 (RCC1) does not appear to be a factor in nuclear reformation^24^. Ran is a small Ras-related GTPase that is known to control the directionality of transport of macromolecules across the NE through the NPCs in addition to functioning in NE formation. RCC1 is a Ran-specific guanine nucleotide-exchange factor, and its enzymatic activity converts RanGDP to RanGTP in the nucleus. RCC1 is located on the chromatin by binding via the nucleosomes; so the presence of RCC1 on DNA implies the presence of a nucleosome structure^27^. Using micrometre-sized beads, it has been proposed that the nucleosome, but not DNA, is a key factor involved in directly assembling the functional NE^28^. Despite the successful achievements of these *in vitro* experiments using *Xenopus* egg extracts, they cannot provide information about spatiotemporal regulation of the molecules involved.

To obtain such spatiotemporal information for the factors required for NE assembly, we have developed new experimental systems using living eggs or embryos. In this study, we used fertilised mouse eggs in which DNA-conjugated beads (hereafter, ‘DNA-beads’) were introduced by microinjection, and live-cell imaging, immunostaining and electron microscopy were used to determine whether such exogenous DNA could induce formation of a functional nucleus in living mouse embryos at early stages of development. This experimental system makes it possible to examine the function of the molecules of interest inside the developing embryos and their spatiotemporal regulation, which was not possible in experiments using egg extracts. Additionally, our imaging technique allows us to monitor cellular responses and the development of embryos up to the blastocyst stage, because it has extremely low toxicity^29^. We evaluated the integrity of nuclei induced artificially on DNA-beads by analysing the accumulation of core histones, nucleosome formation, nuclear membrane assembly, NPC formation and nuclear import activity.

## Results

### Microinjection of DNA-beads into fertilised mouse eggs and their effects on further development

**Figure 1:**
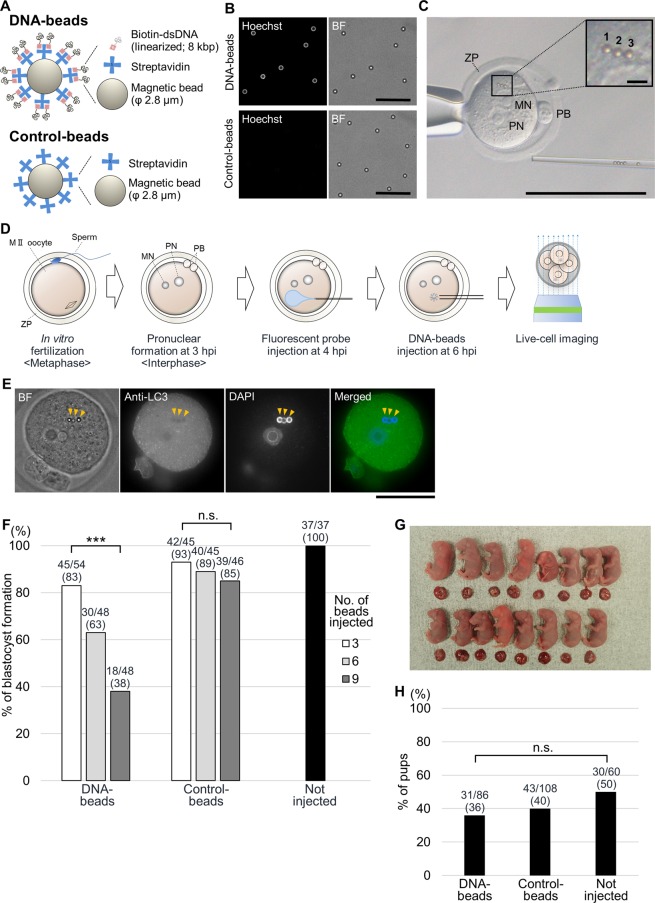
Establishment of bead injection method. (**A**) Schematic diagram of DNA-binding reacted beads (DNA-beads) and negative control beads (control-beads). Biotinylated dsDNA binds to magnetic beads via streptavidin. (**B**) Observation of DNA localization around the DNA-beads and control-beads. The upper left and lower left panels show Hoechst 33342 staining around DNA-beads and control-beads, respectively. The right panels show bright field (BF) images (scale bars = 25 μm). Hereafter, the DNA-beads and control-beads are compared. (**C**) Beads were microinjected into the cytoplasm while avoiding the nuclei and polar bodies. Inset shows the injected beads at a 10× magnification of the boxed region. MN, maternal pronucleus; PN, paternal pronucleus; PB, polar body; ZP, zona pellucida. Scale bars = 100 μm (overview) or 5 μm (enlarged: upper right). (**D**) Experimental diagram. After *in vitro* fertilisation, mouse oocytes arrested at the second meiotic metaphase restart the cell cycle. Maternal and paternal pronuclei are gradually visible after incubation for 3 h. Fluorescent probes were microinjected into the cytoplasm of fertilised eggs at 4 h post-insemination (hpi). Subsequently, DNA- or control-beads were microinjected into the cytoplasm of the fertilised eggs at 6 hpi. The fertilised eggs were observed for 72 h using a confocal laser microscope. Injected probes are listed in Supplementary Table S3. (**E**) Immunostaining for LC3, an autophagy-related protein. From left to right, BF, anti-LC3, DAPI-stained and merged images are shown. The yellow arrowheads show the location of DNA-beads. Scale bar = 50 μm. The numbers of fertilised eggs used for each experiment are listed in Supplementary Table S3. (**F**) Comparison of development rates to the blastocyst stage after bead injection. The grey scale shows the numbers of injected beads: white, grey, dark grey and black indicate 3, 6, 9 and 0 beads, respectively. The beads were microinjected into fertilised eggs (DNA-beads; *P* = 1.19 × 10^−5^, control-beads; *P* = 0.42 by chi-squared test). (**G**) After euthanasia and Caesarean section of the recipient female mice, foetuses were obtained from fertilised eggs injected with DNA-beads. (**H**) Comparison of birth rates between DNA-bead-injected, control-bead-injected and not-injected fertilised eggs (*P* = 0.23 by chi-squared test).

To understand the function of DNA in nuclear formation, DNA-beads were microinjected into fertilised mouse eggs as follows (see Materials and Methods for details). The pGADT7 vector, a dsDNA plasmid with no transcription initiation site (i.e., lacking transcriptional activity), was linearized by digestion with appropriate restriction enzymes, and its 3′-end was labelled with biotin. This biotin-labelled dsDNA was immobilized on streptavidin-coated magnetic beads that had a diameter of approximately 2.8 μm (Fig. 1A)^30^. Streptavidin-coated magnetic beads without dsDNA were used as the negative control (hereafter, ‘control-beads’; Fig. 1A). The DNA- and control-beads were stained with Hoechst 33342, a DNA-specific fluorescent dye, as indicated in Fig. 1B. Beads were introduced into the cytoplasm of *in vitro*-fertilised eggs using a piezo-activated micromanipulator, as used in intracytoplasmic sperm injection (Fig. 1C and Supplementary Movie)^31^.

The integrity of the nuclei formed at the beads was evaluated by immunofluorescence staining, electron microscopy and live-cell imaging. For the live-cell imaging, mRNA encoding fluorescent proteins fused with a nuclear protein and/or fluorescently labelled antigen-binding fragments (Fabs) as a fluorescence probe was first microinjected into fertilised eggs. The fertilised eggs were then injected with DNA-beads and observed for approximately 72 h using spinning-disk confocal fluorescence microscopy (Fig. 1D)^29^. This live-cell imaging technique allowed us to monitor the beads in the embryos until at least the morula stage, and it demonstrated that the beads were randomly distributed in the blastomeres of the morula stage by repeated random distribution in the dividing cells during mitosis.

We first tested whether the DNA-beads microinjected into fertilised eggs would be targeted by autophagy because about 30% of the DNA-beads are targeted and ‘eaten’ by autophagy when introduced into somatic cells using a transfection reagent^30^. To do this, the DNA-beads were stained using indirect immunofluorescence with a specific antibody against LC3, a marker for autophagy. No signals for LC3 were observed around the beads, suggesting that the DNA-beads microinjected into the fertilised eggs were not subjected to autophagy (Fig. 1E).

We next examined the effects of the DNA-beads on embryogenesis. About 83% of the fertilised eggs reached the blastocyst stage when three DNA-beads were introduced per fertilised egg, and this percentage decreased as the numbers of beads increased; in contrast, about 85% of the fertilised eggs injected with control-beads reached the blastocyst stage, even when up to nine beads were introduced (Fig. 1F and Supplementary Table S1). Thus, an increase in the number of DNA-beads resulted in a significant decrease in the percentage of eggs reaching the blastocyst stage. This is probably because the presence of a large number of DNA-beads in the eggs caused the removal of DNA-binding proteins from the native nucleus and/or activation of a cell-cycle checkpoint. We further examined the influence of the DNA-beads on development. When up to three DNA-beads were microinjected into each fertilised egg, no significant impairments to full-term development were observed compared with fertilised eggs injected with control-beads and non-injected control fertilised eggs (Fig. 1G, H and Supplementary Table S2). Based on these results, we decided to inject three DNA-beads into each fertilised egg for further analysis.

### Histone assembly and consequent nucleosome formation around the DNA-beads

**Figure 2:**
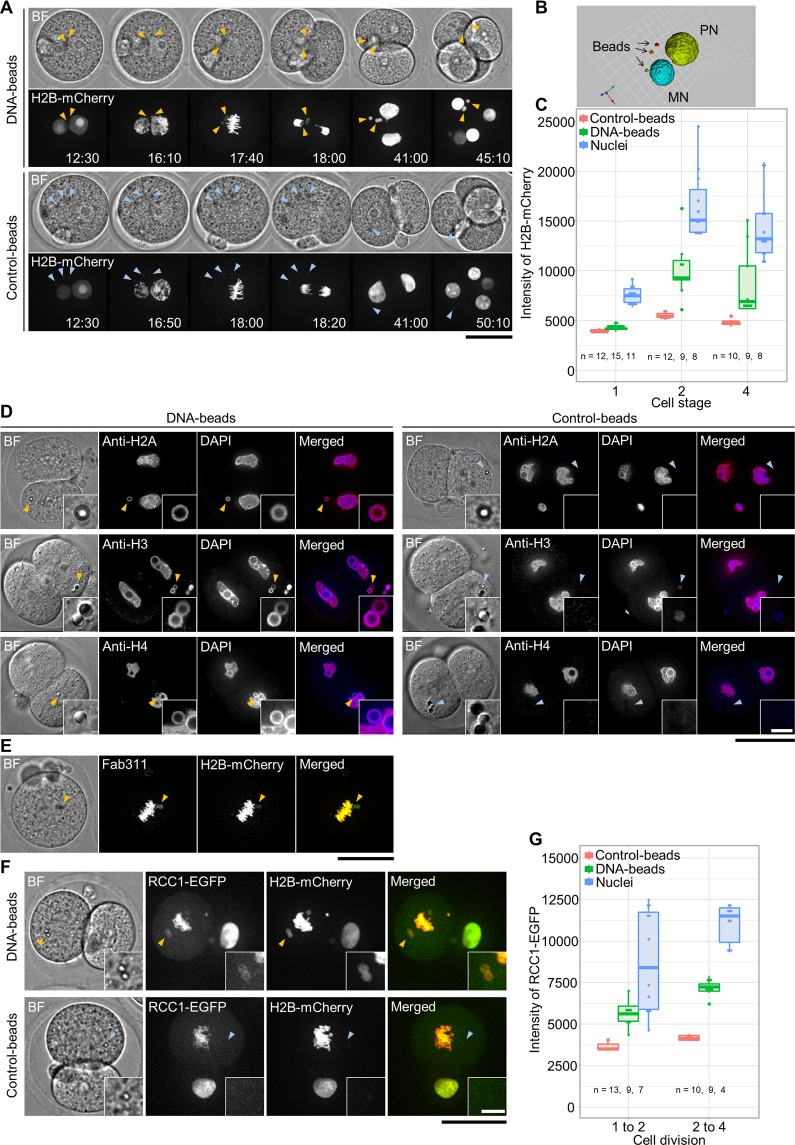
Observation of histones and nucleosomes around DNA-beads. (**A**) Time-lapse images of histone H2B accumulation around DNA-beads and control-beads. In each, the top and bottom panels show bright field (BF) and fluorescence images, respectively. The time course is shown from left to right. Arrowheads show the position of beads. Scale bar = 50 μm. (**B**) Three-dimensional (3D) image reconstruction of nuclei and beads. The brightness of histone H2B-mCherry signals around the DNA-beads was sufficient to form the 3D images. (**C**) The intensity of histone H2B-mCherry signals around the DNA-beads increased during the progression of cell stage. (**D**) Accumulation of histones around the DNA-beads. The DNA-beads and control-beads are compared. In each, embryos immunostained using anti-histone H2A, H3 and H4 antibodies are shown, from top to bottom. From left to right, BF and anti-histone immunostained, DAPI and merged images are shown. Arrowheads show the positions of beads. Scale bars = 50 μm (overview) and 5 μm (enlarged inset). (**E**) Phosphorylation of histone H3 Serine 10 residue around the DNA-beads at the metaphase of first mitosis. From left to right, BF, Fab311 (anti-histone H3 Serine 10), histone H2B-mCherry and merged images are shown. Scale bar = 50 μm. (**F**) The localization of RCC1 around the DNA-beads. The DNA-beads and control-beads are compared. In each row, BF, RCC1-EGFP, histone H2B-mCherry and merged images are shown from left to right. Scale bars = 50 μm (overview) and 10 μm (enlarged: lower right). (**G**) RCC1 signals around the DNA-beads increased during cell division. The numbers of fertilised eggs used for each experiment are listed in Supplementary Table S3.

To evaluate the DNA structures surrounding the microinjected DNA-beads, we examined the assembly of core histones around them. Live-cell imaging revealed that histone H2B fused with mCherry^32^ did not appear on the DNA-beads until the early stage of metaphase of the first mitotic division (12:30-16:10 min in Fig. 2A), but started accumulating around the beads at the metaphase of the first mitotic division (17:40-18:00 min) and peaked at the 2-cell stage (41:00 min). In contrast, no fluorescence signals were observed on the control-beads throughout the observation period (Fig. 2A). Quantification of the amount of histone H2B-mCherry on the DNA-beads using Volocity software confirmed that the accumulation of histone H2B occurred after the first mitotic division (Fig. 2B, C). The assembly of other core histone proteins such as H2A, H3 and H4 was also examined by indirect immunofluorescence staining using specific antibodies. Signals for all core histone proteins tested were detected on the DNA region of the DNA-beads, whereas no signals were detected on the control-beads (Fig. 2D). Because the serine-10 residue of histone H3 in the nucleosome is phosphorylated at metaphase^33^, we tested whether histone H3 on the DNA-beads would also be phosphorylated during mitosis. Monoclonal antibody Fab311, which specifically recognizes a phosphorylated serine-10 residue of histone H3, stained the DNA of the DNA-beads to the same degree as metaphase chromosomes (Fig. 2E), suggesting that the DNA-histone complex on the beads was organized into a metaphase-like chromatin structure. We further tested for the presence of RCC1. This is mainly associated with nucleosomes through interactions with histones H2A and H2B^34^, so an RCC1 signal on the DNA implies the existence of nucleosomes^35,36^. Live-cell imaging of the RCC1-EGFP showed weak but significant signals on the DNA-beads at metaphase or anaphase of the 1-cell stage embryos and increased gradually until the 4-cell stage (Fig. 2F, G). These data suggest that nucleosomes were formed on the exogenous DNA.

### Assembly of nuclear membranes/envelope around the DNA-beads

**Figure 3:**
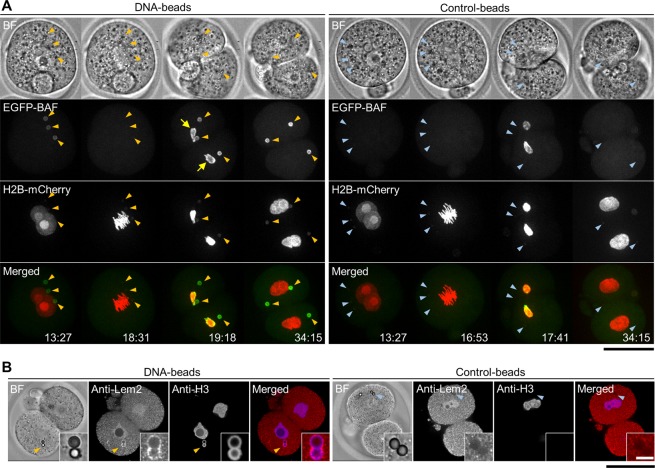
Observation of the nuclear membrane proteins around DNA-beads. (**A**) The fluorescent images of BAF. EGFP-BAF mRNA was injected before bead injection. The DNA-beads and control-beads are compared. In each, the top and bottom panels show bright field (BF) and fluorescence images. The time course is shown from left to right. Arrowheads indicate the position of beads. Arrows indicate the nuclei. Scale bar = 50 μm. (**B**) Images of cells immunostained for Lem2. The DNA-beads and control-beads are compared. In each, BF, anti-Lem2, anti-H3 and merged images are shown, from left to right. Scale bar = 50 μm. The numbers of fertilised eggs used for each experiment are listed in Supplementary Table S3.

We next observed the formation of nuclear membranes. In somatic cells, BAF, a DNA-binding protein, is known to assemble at a distinct region, called the ‘core region’, of telophase chromosomes to form an immobile complex; this BAF-dependent structure facilitates NE reformation by directly binding NE proteins including LEM-domain proteins such as emerin^16,19,20^. In embryos, intense BAF signals were observed on the telophase chromosomes of the first mitotic division (19:18 min in the left panels of Fig. 3A). Within the same embryos, BAF was also observed around the DNA-beads (arrowheads in the left panels in Fig. 3A), whereas no signals were observed around the control-beads (arrowheads in the right panels of Fig. 3A). Indirect immunofluorescence staining using a specific antibody to the NE protein Lem2 showed positive signals, suggesting that Lem2 was recruited to the DNA-beads (Fig. 3B). Under these conditions, the fluorescence signal of EGFP-BAF was not prominent in interphase nuclei (34:15 min), possibly because of the presence of many interacting factors in the cytoplasm and fewer in the nucleus in fertilised mouse eggs/embryos.

**Figure 4:**
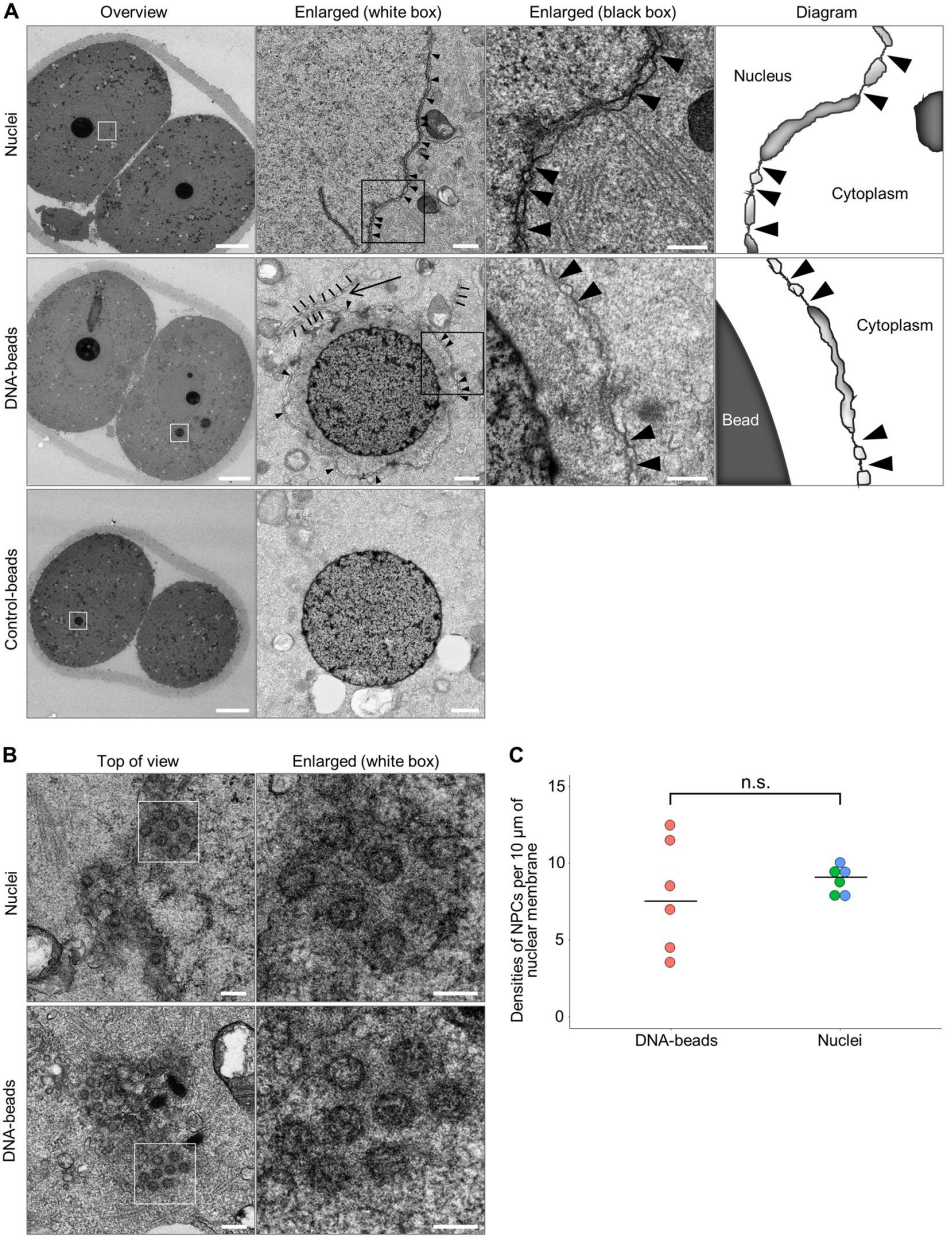
Observation of nuclear membranes and pore structures around DNA-beads. (**A**) Electron microscopy images of nuclei, DNA-beads and control-beads. In each, overview, enlarged images (white boxes in the overviews), more highly enlarged images (black boxes) and a schematic diagram are shown from left to right. Arrowheads show the nuclear pore-like structures on the nuclear membrane-like structure. Scale bar = 10 μm, 0.5 μm and 0.2 μm, from left to right. (**B**) Electron microscopy images showing a top view of the pore-like structures around nuclei and DNA-beads. Scale bar = 0.2 μm (top of view) and 0.1 μm (white box in overview). (**C**) Comparison of the densities of pore-like structures on the membranes on DNA-beads and native nuclei. Electron microscopy images were obtained with Z-slices (thickness 5 μm) and membrane lengths, and the densities of pore-like structures were counted in each x-y plane. Then, the numbers were normalized against the length of membrane, and indicated as the values per 10 μm nuclear membrane. Red, green and blue dots show the densities of pores on the DNA-beads, native nuclei of embryos injected with DNA-beads, and native nuclei of embryos injected with control-beads, respectively. There was no significant difference in the distribution between DNA-beads and native nuclei (*P* = 0.56 by Wilcoxon rank sum test).

Electron microscopy of the DNA-beads showed that they were surrounded by double-membrane structures resembling the native NE (compare the top panels with the middle panels in Fig. 4A). Additionally, precursor membranes of the NE, called ‘annulate lamellae’^37^, lay near the DNA-beads and were at least partly fused to the nuclear membrane-like structures (see the large arrow in Fig. 4A), suggesting that this membrane structure was the NE. Interestingly, nuclear pores and nuclear pore complex-like structures were observed on the membrane structure (see arrowheads in Fig. 4A). The top view shows that the NPC-like structures assembled around the DNA-beads were remarkably similar to those of native nuclei (Fig. 4B). To evaluate the NPC-forming activity of the membranes, we determined the densities of NPC-like structures on the DNA-beads and compared these values with those of native nuclei. There was no significant difference between the DNA-beads and the native nuclei (Fig. 4C). It should be noted that the densities of NPCs around the DNA-beads were more variable than those of the native nuclei (Fig. 4C), suggesting that the position of the DNA-beads within the embryos might cause variations in NPC formation. We also determined the densities of the NPC-like structures on the DNA-beads located away from the native nuclei but near the cell cortex (Fig. S1). Electron microscopy showed that the densities of these NPCs were much lower than those of the native nuclei (Fig. S1A, B). These results suggest that the positioning of the DNA-beads within the embryos is important in determining NPC formation. In contrast, no nuclear membrane assembly or nuclear pore-like structures were found around the control-beads (Fig. 4A). These results suggest that DNA can mediate autonomous assembly of nuclei or nucleus-like structures in living mouse embryos.

### Integrity of the NE and NPCs around DNA-beads

**Figure 5:**
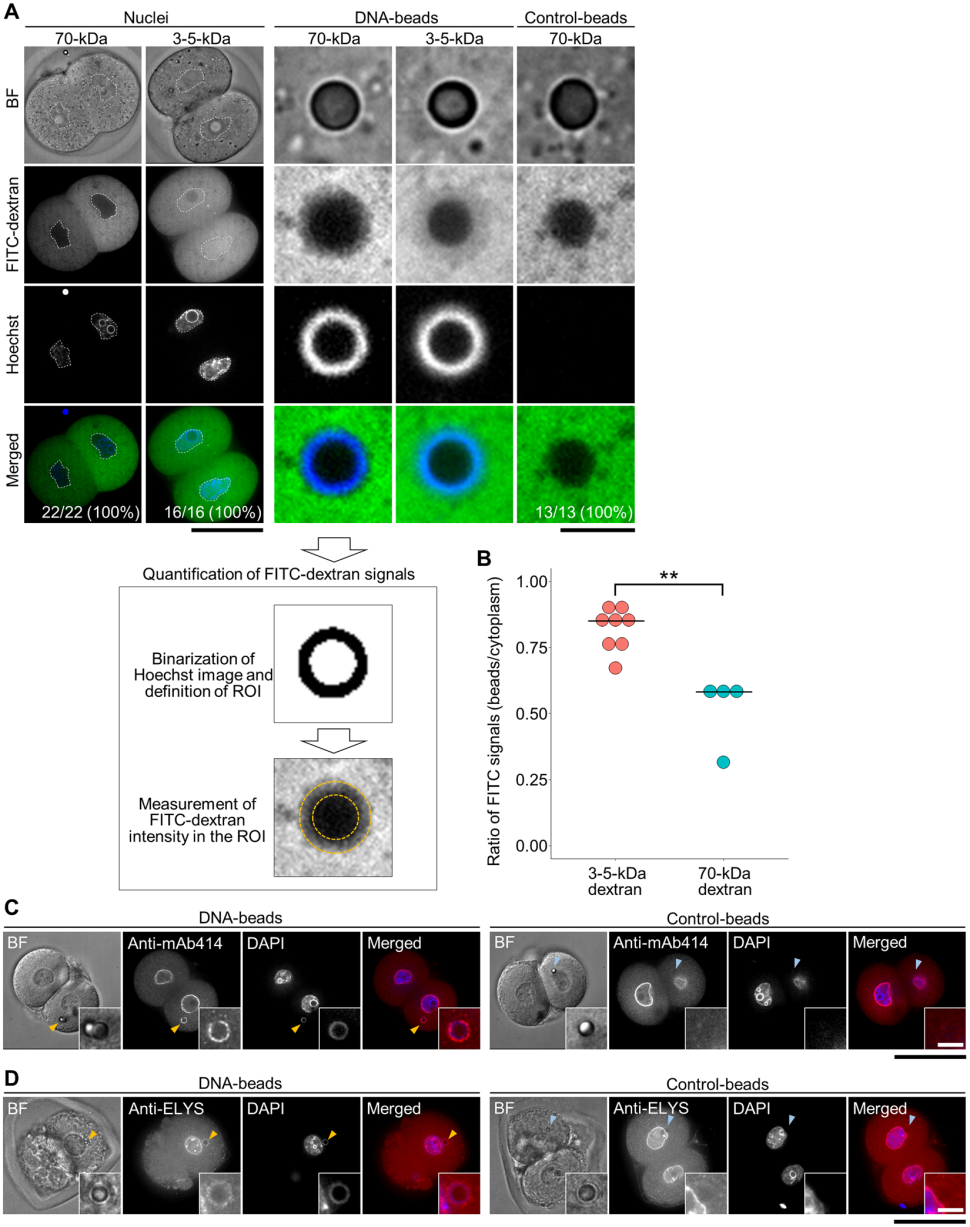
Nuclear membrane-like structures covered the entire DNA-beads and molecules entered selectively by size. (**A**) Comparison of the permeation of different-sized molecules between DNA-beads and control-beads. Dextran-FITC (70-kDa and 3-5-kDa) was microinjected into cytoplasmic regions of 2-cell embryos and fluorescence was observed. In each, bright field (BF), dextran-FITC, Hoechst 33342 DNA staining and merged images are shown from top to bottom. Dotted lines show the nuclei. Scale bars in the panels showing nuclei and beads are 50 μm and 5 μm, respectively. The lower part of the figure represents the method for the quantification of dextran-FITC signals. (**B**) Quantification of the dextran-FITC signals around the DNA-beads. The values are expressed as the ratio of the FITC signal of the region of interest to that of the cytoplasmic region. (*P* = 0.004 by Wilcoxon rank sum test). Images of cells immunostained using mAb414 (**C**) and anti-ELYS (**D**) to localize NPCs. The DNA-beads and control-beads are compared. In each, BF, antibody-stained, DAPI and merged images are shown from left to right. Arrowheads show the position of beads. Scale bar = 50 μm. The numbers of fertilised eggs used for each experiment are listed in Supplementary Table S3.

One of the functions of the NE is to isolate the nucleus spatially from the cytoplasm, because it can exclude macromolecules larger than 50-60 kDa via the function of the NPCs. To evaluate the integrity of the NE formed around the DNA-beads, we examined the exclusion activity of the NPCs by observing the behaviour of 70-kDa or 3-5-kDa dextran labelled with fluorescein isothiocyanate (FITC) microinjected into embryos that had been preinjected with DNA-beads (Fig. 5A). The 70-kDa FITC-dextran was excluded from entry to the nuclei while the 3-5-kDa dextran was diffusely located within them (left panels of Fig. 5A), suggesting that this technique could be used to evaluate NE integrity. As with the nuclei, the fluorescence signal of 70-kDa dextran associated with the DNA-beads was predominantly excluded from the spherical regions of the beads, while that of 3-5-kDa dextran was not (middle panels of Fig. 5A). Quantification of the fluorescence signals showed significantly greater exclusion of 70-kDa dextran from the DNA-beads compared with that of 3-5-kDa (Fig. 5B). 250-kDa dextran produced similar results to 70-kDa dextran (Fig. S2). These results suggest that DNA-beads were enveloped with an NE-like structure and that the reconstituted NPCs could exclude macromolecules similarly to embryonic nuclei.

The presence of NPC component proteins was examined by indirect immunofluorescence staining using the monoclonal antibody mAb414, a poly-specific antibody to FG-bearing nucleoporins (FG-Nups) and a specific antibody against ELYS, a component of the outer ring structure of the NPC. Fluorescence signals for the FG-Nups and ELYS were detected around the DNA-beads (Fig. 5C, D) but not around the control-beads (Fig. 5C, D). These data suggested that NPC components, at least in part, were integrated into the nuclear pore-like structures of the NE around the DNA-beads.

We also examined NPC assembly around DNA-beads that were located distant from the native nuclei by indirect immunofluorescence staining using mAb414. The signals observed were weaker than those near the native nuclei (Fig. 5C and S1C), supporting our hypothesis that spatial positioning is a key factor in nuclear formation.

### Functional analysis of nuclear pore-like structures formed around DNA-beads

**Figure 6:**
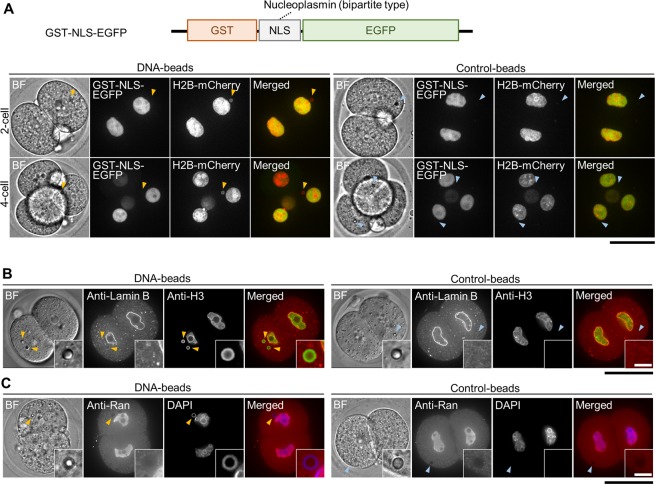
NPCs around DNA-beads did not exhibit nuclear transport. (**A**) The top drawing shows the construction of GST-NLS-EGFP; this NLS is a bipartite NLS originating from nucleoplasmin^19^. Images show fluorescence images of 2- and 4-cell stage embryos expressing GST-NLS-EGFP around DNA-beads and control-beads. In each, BF, GST-NLS-EGFP, histone H2B-mCherry and merged images are shown from left to right. Upper and lower panels show 2-cell stage and 4-cell stage embryos, respectively. Scale bar = 50 μm. (**B**) Images of cells immunostained for lamin B, a structural component of the nuclear lamina around DNA-beads and control-beads. In each, BF, anti-lamin B, anti-H3 and merged images are shown from left to right. Scale bar = 50 μm. (**C**) Images of cells immunostained for Ran. In each, BF, anti-Ran, DAPI and merged images are shown from left to right. Scale bar = 50 μm. The numbers of fertilised eggs used for each experiment are listed in Supplementary Table S3.

We used live-cell imaging to examine the nuclear import activity of the NE-like structures formed at the DNA-beads from the 1-cell to the 8-cell stage. For this purpose, we used a fluorescence probe for a nuclear localization signal (NLS) fused with glutathione S-transferase (GST), and EGFP (GST-NLS-EGFP) at its N-terminus and C-terminus, respectively, to avoid any false positive signals arising from molecular diffusion through the nuclear pore (Fig. 6A). A bipartite NLS sequence derived from nucleoplasmin was used as an NLS^38^. Although fluorescence signals were detected in the native nuclei, they were not detected on the DNA-beads at any stage tested (Fig. 6A) or on the control-beads (Fig. 6A). Similar results were obtained when EGFP-tagged monopartite NLS (EGFP-NLS)^39^ derived from methyl-CpG-binding domain protein 1 (MBD1) was used as an NLS (Fig. S3). To further examine the nuclear import activity for endogenous proteins, the embryos were stained with an anti-lamin B antibody. Lamin B is a nuclear structural protein that constitutes the nuclear lamina and is known to be transported into the nucleus by function of its NLS and the nuclear transport machinery. Although lamin B signals were found in the native nucleus as expected, no signals were found around the DNA-beads (Fig. 6B), suggesting that the NE and the NPCs on the beads have incomplete function in terms of macromolecular transport.

This impaired nuclear import activity in the DNA-bead-mediated nuclei permitted us to analyse the behaviour of the proteins required for nuclear import. Ran, a small Ras-related GTPase, controls the directionality of transport of macromolecules across the NE through the NPCs^8,40,41^ and has a role in the assembly of the NE^24^. Indirect immunostaining analysis using an anti-Ran antibody showed that no fluorescence signals for Ran could be detected around the DNA-beads or control-beads, although signals were detected in the native nuclei at the 2-cell stage (Fig. 6C). This suggested that lack of Ran on the DNA-beads might have been a cause of the impaired import activity of the DNA-bead-mediated nuclear structures.

**Figure 7:**
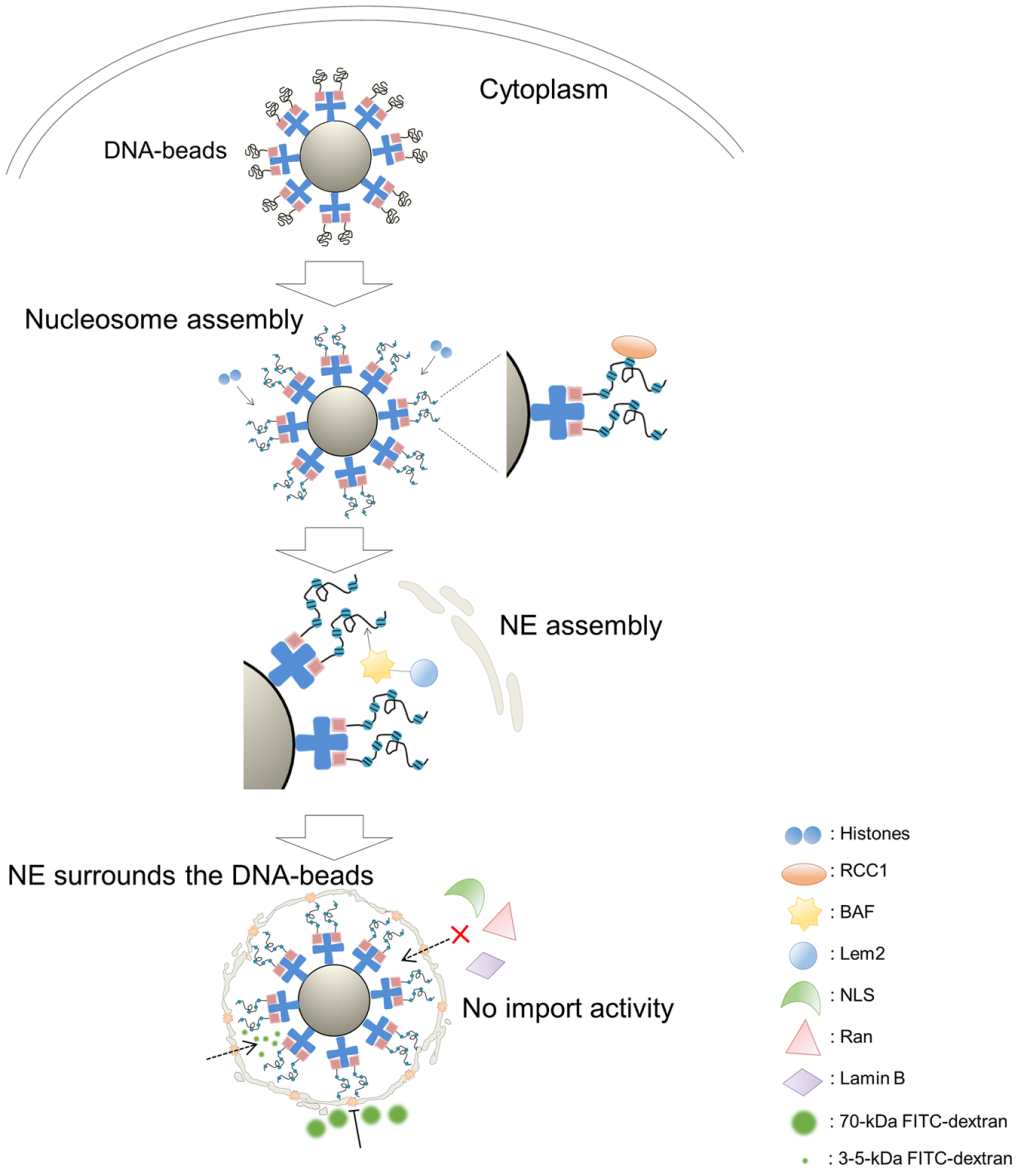
Model of DNA-bead-mediated membrane and pore structure, and its function. Marks are listed at bottom right. Histones and RCC1 accumulate around DNA-beads in the cytoplasm and BAF/Lem2, which is directly involved in NE formation, accumulates around the DNA-beads. Freely diffusing molecules are selectively permeable by size, but no nuclear import activity is observed in the reconstructed ‘nucleus’.

In summary, nucleosome structures, nuclear membrane-like structures and partial components of nuclear pore-like structures formed around DNA-beads in fertilised mouse eggs. However, these structures had no detectable nuclear import function, probably because of the absence of Ran around the DNA-beads (Fig. 7).

## Discussion

The earliest attempt to form artificially-induced nuclei in living organisms involved the injection of bacteriophage λ DNA into unfertilised *Xenopus* eggs^42^. In those experiments, electron microscopy detected a double-membrane structure containing NPCs around the injected DNA. Immunofluorescence staining also showed the presence of nuclear protein lamins underneath the inner membrane of the double-membrane structure, similar to those present in the native NE, indicating that the nuclear import machinery had been established in the reconstructed nucleus. Our results here partly reproduced these findings, in that DNA microinjected into the cytoplasm could constitute NE-like structures in mouse cleavage-stage embryos. However, our results showed one clear difference from that pioneering study, in that no nuclear transport activity was found in the DNA-bead-induced artificial nuclei (Fig. 6). This difference might have arisen because of the different conditions used.

One of these differences was our use of fertilised rather than unfertilised eggs as recipients. Heald *et al*. have reported successful formation of nuclei with import activity on DNA-beads in extracts of fertilised *Xenopus* eggs; however, they used DNA-beads that had been preincubated in “unfertilised” *Xenopus* egg extracts^23^; this suggests that fertilisation may reduce the nucleus-forming activity. It is well known that fertilisation causes the release of Ca^2+^ from its cytoplasmic stockpile to generate repeating waves of Ca^2+^ over the surface of fertilised eggs. These repeating Ca^2+^ waves activate many cellular events, such as the resumption of meiosis and consequent reorganisation of the nuclear and chromatin structures^43,44^. Our previous observations using live-cell imaging demonstrated that in mouse oocytes, such Ca^2+^ spikes ended within 3 h after sperm entrance^45^. The most prominent changes in the nuclear and chromatin structures at that time are decondensation of the sperm chromatin and pronuclear formation. Immediately after sperm entrance, sperm-specific DNA binding proteins, i.e., protamines, are removed and maternally-stored histones are incorporated into the male pronuclear chromatin with the help of histone chaperones^46^. Thus, histone H3.3, a replication-independent chromatin-loading H3 variant, is incorporated into the male pronucleus with the help of the male chromatin-specific histone chaperone, HIRA protein^47^. Inoue and Zhang have reported that in mice, H3.3 incorporation into the sperm chromatin starts immediately after sperm-egg fusion and is complete within 2 h^35^. They also found that the formation of nuclear laminae highlighted by lamin B1 was seen in male and female pronuclei from 4 h after sperm entry (fertilisation)^35^. Thus, fertilisation causes quantitative and qualitative differences in the nuclear and chromatin structures and functions of unfertilised and fertilised eggs. For technical reasons, we injected DNA-beads 6 h after insemination, at which time the intrinsic nuclear and chromatin reorganisation processes were complete (Fig. 1D). This means that in our experiments we used a fertilised egg whose nucleation ability had weakened. Therefore, we consider that this difference in the timing of bead injection may be one of the causes of the differences between our results and those of previous studies.

In addition to histones and chaperones, it has been reported that Ran changes its localization dynamically in a cell-cycle-dependent manner during mouse oocyte maturation and subsequent fertilisation. Thus, during meiotic divisions, it is concentrated at the meiotic spindle microtubules and then after sperm-egg fusion, it disperses throughout the cytoplasm at the time of extrusion of the second polar body, and is concentrated gradually in the male and female pronuclei thereafter^48^. This dynamic change in Ran localization following fertilisation suggests that the timing of injection might be important for nuclear formation at the DNA-beads. Other factors required for NE assembly might also change their localization following fertilisation of eggs.

Another difference between our results and those reported previously by Forbes and her colleagues^42^ concerning the nuclear import ability of the reconstructed nucleus might have arisen from the length and/or form of the DNA used as a source. We used a relatively short (~8 kbp) linearized DNA attached to the beads, whereas Forbes *et al*. used a longer (~49 kbp) and aggregated bacteriophage λ DNA^42^. One of the noteworthy findings of our study was that core histones were recruited to the DNA around the beads and that nucleosomes were formed (Fig. 2). Because nucleosomes are arranged sequentially on the chromatin in units of 146 bp DNA^2^, it is possible that quantitative, as well as qualitative, differences in the structure and function of nucleosomes may have arisen between our DNA and that used by Forbes *et al*.^42^. Nucleosomes are now known to be required for the assembly of NPCs with nuclear import activity^28,35^. This suggests that the DNA-beads in our experiments did not contain sufficient nucleosomes to form functional NPCs, unlike those with bacteriophage DNA used by Forbes *et al*.^42^. This idea is supported by the relatively low intensity of RCC1-EGFP on the DNA-beads compared with that in the native nuclei (Fig. 2G).

Lack of Ran on the DNA-beads also helps explain the defects in nuclear transport activity of the DNA-bead-induced nuclear structures (Fig. 5B). Ran is well known to be required for nuclear transport activity^8,49^. The GTP-bound form of Ran (RanGTP) accumulates in the nucleus by the function of RCC1, while the GDP-bound form of Ran (RanGDP) accumulates in the cytoplasm by the function of the Ran activating protein, RanGAP1. Consequently, the RanGTP/RanGDP gradient formed between the nucleus and the cytoplasm determines the direction of molecular transport^50–52^. Therefore, nuclear transport could not occur in the DNA-bead-induced nuclear structures that lacked Ran molecules, probably because of the dynamic changes of Ran localization during fertilisation, described above.

The spatial position of the DNA-beads introduced to the fertilised eggs might also have modulated the subsequent fate and capability of the DNA-beads. Thus, we found differences between the NE structures in the DNA-beads located near the cortex of the embryo and those near the centre close to the native nucleus. The DNA-bead-induced artificial nuclei near the cortex contained no or few NPCs, whereas those close to the native nuclei contained many (Figs. 4 and S1). This suggests that spatial position is another determinant of NE assembly.

Our approach to inducing artificial nuclei in living mouse embryos, as presented here, has advantages for understanding the factor(s) and spatiotemporal regulation involved in forming normal nuclei. To this end, we injected DNA-beads into living fertilised mouse eggs to induce the formation of artificial nuclei. Surprisingly, the fertilised eggs carrying the DNA-beads were viable and gave rise to normal pups. This indicates that the presence of several such beads at the 1-cell stage did not impair subsequent embryonic development. Thus, our experimental system is appropriate for evaluating the normal processes of cell division during development under normal physiological conditions. In such *in vivo* conditions, quantitative information can be obtained in addition to the spatiotemporal information obtained *in vitro*. Finally, the accumulation of knowledge about mammalian cells using widely-available mouse models may be important for understanding the general mechanisms of nuclear formation.

## Materials and Methods

### Antibodies

Commercially available antibodies against the following proteins were used in this study. Mouse monoclonal antibodies against histone H2A (Medical & Biological Laboratories Co., Ltd., MBL, Nagoya, Japan; D210-3); histone H3 (Monoclonal Antibody Laboratory Inc., Iida, Japan; MABI0301); histone H4 (Monoclonal Antibody Laboratory Inc.; MABI0400); FG-Nups of the NPC (mAb414, BioLegend, San Diego, CA, USA; 902901); ELYS (Abcam, Cambridge, UK; ab14431); Ran (BD Biosciences, Franklin Lakes, NJ, USA; BD610341); and Alexa Fluor 488-labelled mouse-anti-histone H3 Ser10phospho Fab fragment (a gift from Hiroshi Kimura)^33^. Rabbit polyclonal antibodies against LC3 (MBL, PM036), LEM domain-containing protein 2 (Atlas Antibodies, Bromma, Sweden; HPA017340) and lamin B1 (Santa Cruz Biotechnology, Inc., Dallas, TX, USA; sc-20682). CF 555-conjugated goat anti-mouse IgG (H + L) secondary antibody, CF 488-conjugated goat anti-mouse IgG (H + L) secondary antibody, CF 555-conjugated goat anti-rabbit IgG (H + L) secondary antibody and CF 488-conjugated goat anti-rabbit IgG (H + L) secondary antibody were all purchased from Nacalai Tesque Inc., Kyoto, Japan (20231-1, 20302-1, 20232-1 and 20019-1, respectively).

### DNA-beads

DNA-beads were prepared as described^30^. Briefly, the pGADT7 vector (8.0 kbp; Clontech Laboratories, Inc., Mountain View, CA, USA; 630442) was digested using *Cla*I and *EcoR*I restriction enzymes, and its 3′-end was labelled with biotin. Then, the larger fragment, including biotinylated dsDNA, was purified and immobilized on Dynabeads M-270 Streptavidin (Thermo Fisher Scientific, Waltham, MA, USA). To confirm DNA binding to beads, DNA-beads and control-beads were stained with 10 μg/mL Hoechst33342 (Dojindo Laboratories, Kumamoto, Japan; 23491-52-3) for 15 min, and observed using a CV1000 fluorescence microscope (Yokogawa Electric Corporation, Tokyo, Japan).

### Animals

This study conformed to the requirements of the Guide for the Care and Use of Laboratory Animals. All animal experiments were approved by the Animal Care and Use Committee at the Research Institute for Kindai University (permit number: KABT-28-001). ICR strain mice (11-16 weeks old) were obtained from Japan SLC, Inc. (Shizuoka, Japan). Room conditions were standardized with temperature maintained at 23 °C, relative humidity at 50% and a 12/12 h light/dark cycle. Animals had free access to water and commercial food pellets. Mice used for experiments were euthanized by cervical dislocation.

### *In vitro* fertilisation

Superovulation was induced in female ICR mice (11-16 weeks old) by intraperitoneal injections of 10 IU pregnant mare serum gonadotropin (PMSG, ASKA Animal Health Co., Ltd., Tokyo, Japan) and 10 IU human chorionic gonadotropin (hCG, ASKA Animal Health) at 48 h intervals. Cumulus-intact oocytes were recovered at euthanasia 15-17 h after hCG injection; these oocytes were arrested at metaphase II of the meiotic cell cycle. Spermatozoa were collected from the cauda epididymis of male ICR mice (11-16 weeks old) in 0.2 mL droplets of TYH medium^53^ and capacitated by incubation for 1.5 h at 37 °C under 5% CO_2_ in humidified air. Cumulus-intact oocytes were collected in 0.2 mL of TYH medium and inseminated with spermatozoa (final concentration 75/100 μL); upon insemination, oocytes arrested at metaphase II restarted the meiotic cell cycle and proceeded to interphase in ~3 h. After 1.5 h incubation at 37 °C under 5% CO_2_ in humidified air, the cumulus cells were dispersed by brief treatment with hyaluronidase (Type I-S, 120-300 units/mL; Sigma-Aldrich, St Louis, MO, USA).

### Preparation of mRNAs to express proteins of interest

After linearization of the template plasmids (pcDNA3.1-poly(A)) at the *Xba*I (EGFP-NLS) or *Xho*I (histone H2B-mCherry, EGFP-BAF, RCC1-EGFP and GST-NLS-EGFP, respectively) sites, mRNA was synthesized using RiboMAX^TM^ Large Scale RNA Production Systems-T7 (Promega, Madison, WI, USA), as described^54^. For efficient translation of the fusion proteins in embryos, the 5′-end of each mRNA was capped using Ribo m^7^G Cap Analog (Promega), according to the manufacturer’s protocol. To circumvent integration of template DNA into the embryonic genome, the reaction mixtures for *in vitro* transcription were treated with RQ-1 RNase-free DNase I (Promega). Synthesized mRNAs were treated with phenol-chloroform to remove protein components. The mRNAs were further purified by filtration using MicroSpin^TM^ S-200 HR columns (Amersham Biosciences, Piscataway, NJ, USA) to remove unreacted substrates (RNA reaction intermediates) and then stored at −80 °C until use.

### Probe injection

Probe injection into fertilised eggs was performed as described^54^. Briefly, mRNAs were diluted to 5 ng/μL each and fluorescently labelled Fab fragments were diluted to 15.6 μg/mL using ultrapure water (Thermo Fisher Scientific Barnstead Smart2Pure), and an aliquot was placed in a micromanipulation chamber. Fertilised eggs (approximately 4 h after insemination) were transferred to HEPES-buffered Chatot-Ziomek-Bavister (CZB) medium^55^ in the chamber and injected with mRNA using a piezo-driven manipulator with a narrow glass pipette (1 μm diameter). Once the mRNA solution had been aspirated into the pipette, piezo pulses were applied to the fertilised eggs to break the zona pellucida and plasma membrane. A few picolitres of solution were introduced into the cytoplasm, and the pipette was removed gently. The mRNA-injected fertilised eggs were incubated at 37 °C under 5% CO_2_ in air for at least 2 h before bead injection to wait for protein production.

### Live-cell imaging and image analysis

The fertilised eggs were transferred to 5 μL droplets of KSOMaa including polyvinyl alcohol (PVA; Sigma-Aldrich, P8136-250G) and EDTA on a film-bottomed PS dish (Matsunami Glass Industries, Ltd., Osaka, Japan). Fertilised eggs were imaged two- (2D) and three-dimensionally (3D) using a spinning-disk confocal fluorescence microscopy system (CV1000, Yokogawa Electric Corp.) set at 37 °C in 6% CO_2_, 5% O_2_ and 89% N_2_ with saturated humidity. To prevent the slippage of fertilised eggs during imaging, they were attached to the bottom of the dish by adding PVA to the KSOMaa medium (0.00025%). Images were taken at 3-10 min intervals for 3-4 days under the following conditions: EGFP-labelled (EGFP-BAF, RCC1-EGFP, NLS-EGFP, GST-NLS-EGFP and fluorescently labelled Fab fragments) and mCherry-labelled (histone H2B-mCherry) images were taken at excitation wavelengths of 488 nm and 561 nm, emission wavelengths of 525/50 and 617/73, and laser power from 0.05 to 0.10 mW, respectively. The exposure time was 100 msec and gain was 100%. Forty-six images were taken in the Z-axis at 2.0 μm intervals. The 2D/3D images were constructed using Volocity software (PerkinElmer, Inc., Waltham, MA, USA) and MetaMorph software ver. 7.7.10 (Molecular Devices, San Jose, CA, USA). Image analysis was performed as described^56^.

### Intracytoplasmic bead injection

Fertilised eggs preinjected with mRNAs were transferred to a droplet (about 10 μL) of HEPES-CZB medium in the chamber. Aliquots of about 0.5 μL of beads in ultrapure water were mixed with 10 μL of HEPES-CZB medium containing 3% (w/v) polyvinylpyrrolidone K-90 (PVP K-90) (Nacalai Tesque, 28315-72) in micromanipulation chambers. Some beads were drawn into the glass micropipette (~3 μm diameter) before microinjection. Then, the glass micropipettes with beads were inserted into the cytoplasm of fertilised eggs through the zona and plasma membrane using a piezo micromanipulator. Beads were then introduced into the cytoplasm.

### Dextran injection

Aliquots of 250-kDa dextran conjugated with FITC (FITC-250-kDa-dextran; Sigma-Aldrich, FD250S-100MG), 70-kDa dextran conjugated with FITC (FITC-70-kDa-dextran; Sigma-Aldrich, FD70S-100MG) and 3-5-kDa dextran conjugated with FITC (FITC-3-5-kDa-dextran; Sigma-Aldrich, FD4-100MG) were diluted to 12.5 mg/mL using ultrapure water (Thermo Scientific Barnstead Smart2Pure). Each solution was microinjected into the cytoplasm of both cells of 2-cell stage embryos, which had been preinjected with DNA-beads or control-beads at the 1-cell stage, using a piezo-activated manipulator with a narrow glass micropipette (1 μm diameter). DNA in the embryos was stained with 10 μg/mL Hoechst 33342 for 30 min. For the quantification of the fluorescence signals around DNA-beads, ImageJ software (http://rsb.info.nih.gov/ij/) was used. We first define the region of interest (ROI) to be quantified by converting the Hoechst 33342-stained image (DNA region) to the binary images using the threshold algorithm “Intermodes”. The FITC signals in the ROI and a cytoplasmic region of the same size were measured. The values were expressed as the ratio of the FITC signal of the ROI to that of the cytoplasmic region.

### Embryo transfer

Each group of blastocysts carrying DNA-beads or control-beads was transferred to the uteri of day 2.5 pseudo-pregnant female mice (the numbers of transferred embryos carrying DNA-beads, control-beads and those not injected were *n* = 86, 108 and 60, respectively). Recipient female mice were euthanized at 18.5 days post copulation to evaluate the ability of embryos injected with DNA-beads to develop to full term.

### Immunofluorescence staining

The anti-NPC antibody (mAb414) and anti-ELYS antibody were used in 2-cell stage embryos fixed with ice cold methanol (Nacalai Tesque, 21915-93) for 15 min, and an anti-lamin B1 antibody was used in 2-cell stage embryos fixed with periodate-lysine-paraformaldehyde fixative^57^ for 2 h. The other antibodies were used in pronuclear- and 2-cell stage embryos fixed with 4% paraformaldehyde (Wako Pure Chemical Industries, Osaka, Japan; 163-20145) containing 0.1% PVA for 30 min at 4 °C. After fixation, the embryos were washed twice with phosphate-buffered saline (PBS) containing 0.1% PVA. The embryos were permeabilized with 0.25% Triton X-100 in PBS for 20 min at 4 °C, washed twice with PBS containing 3% bovine serum albumin (BSA) and stored for 60 min or overnight in PBS containing 3% BSA at 4 °C for blocking. The embryos were then incubated with primary antibodies dissolved in PBS containing 1% BSA at 4 °C for at least 1 h. After being washed twice in PBS containing 1% BSA (without or with 0.05% Tween-20), the embryos were further incubated with secondary antibodies for 60 min at 4 °C. Following two washes with PBS containing 1% BSA (without or with 0.05% Tween-20), DNA was stained with 4′,6-diamidino-2-phenylindole (DAPI; 10 μg/mL; Roche Diagnostics, Rotkreuz, Switzerland; 10236276001) for 30 min at 4 °C. The embryos were then observed using a spinning-disk confocal fluorescence microscope system (CV1000) or CSU-W1 (Yokogawa Electric Corp., Tokyo, Japan) equipped with an inverted microscope (Olympus IX-71, Tokyo, Japan) and an electron-multiplying charge-coupled device (EM-CCD) camera (iXON3 DU897E-CS0-#BV-Y, Andor Technology Ltd., Belfast, UK). Three-colour fluorescence images in 140-170 different focal planes at 0.5 μm intervals were captured using a 100× objective lens (UPLSAPO100XS, NA = 1.35) with 407, 488 and 561-nm laser lines, using MetaMorph software version 7.7.10 (Molecular Devices).

### Electron microscopy

Two-cell-stage embryos were fixed with 2.5% (w/v) glutaraldehyde for 2 h and washed twice in 0.1 M PBS (pH 7.4) containing 0.5 mg/mL PVP (Sigma-Aldrich; 9003-39-8). The fixed embryos were embedded in 50 μL droplets of 0.5% agarose (SeaPlaque™ Agarose, Lonza, Basel, Switzerland; 50101) containing 0.5 mg/mL PVP (Sigma-Aldrich; 9003-39-8) on a glass-bottomed microwell dish (Mat Tek Corp., Ashland, MA, USA; P35G-1.5-10-C) and centrifuged at 2000 rpm for 2 min. The embryos were post-fixed with 1% OsO_4_ (3002; Nisshin EM, Tokyo, Japan), stained with 2% (w/v) uranyl acetate (8473-1M; Wako Pure Chemical Industries) for 1 h, dehydrated and embedded in Epon812 (T024; TAAB Laboratory Equipment, Ltd., Reading, UK). Ultra-thin sections (80 nm thick) were prepared using an ultramicrotome (Leica Microsystems, Wetzlar, Germany) and stained with 4% uranyl acetate, followed by a commercial ready-to-use solution of lead citrate (18-0875-2; Sigma-Aldrich). Electron microscope (EM) images were acquired using a JEM-1400 electron microscope (80 kV; JEOL, Tokyo, Japan).

### Statistical analysis

Chi-squared tests and Wilcoxon rank sum tests were performed using custom R programs. *P*-values > 0.05 were considered not significant (n.s.), whereas *P*-values < 0.05 (*), <0.01 (**) and <0.001 (***) were considered significant.

## Supplementary information

Supplementary materials

Supplementary Movie
