# Supplementary material for: Nuclear formation induced by DNA-conjugated beads in living fertilised mouse egg
Source: Sci Rep. 2019 Jun 11;9:8461. doi: 10.1038/s41598-019-44941-6 (PMC6560220; doi:10.1038/s41598-019-44941-6)
Supplement: Supplementary file 1 — Supplementary materials [file 41598_2019_44941_MOESM1_ESM.pdf]

Supplementary Materials for

Nuclear formation induced by DNA-conjugated beads in living fertilised mouse egg

Yuka Suzuki[1], Şükriye Bilir[2,3], Yu Hatano[1], Tatsuhito Fukuda[1], Daisuke Mashiko[1], Shouhei Kobayashi[2], Yasushi Hiraoka[2], Tokuko Haraguchi[3]*, Kazuo Yamagata[1]*

*Correspondence should be addressed to Tokuko Haraguchi (e-mail: tokuko@nict.go.jp) or Kazuo Yamagata (e-mail: yamagata@waka.kindai.ac.jp).

This file includes:

Supplementary Figures S1-S3
Supplementary Tables S1-S3

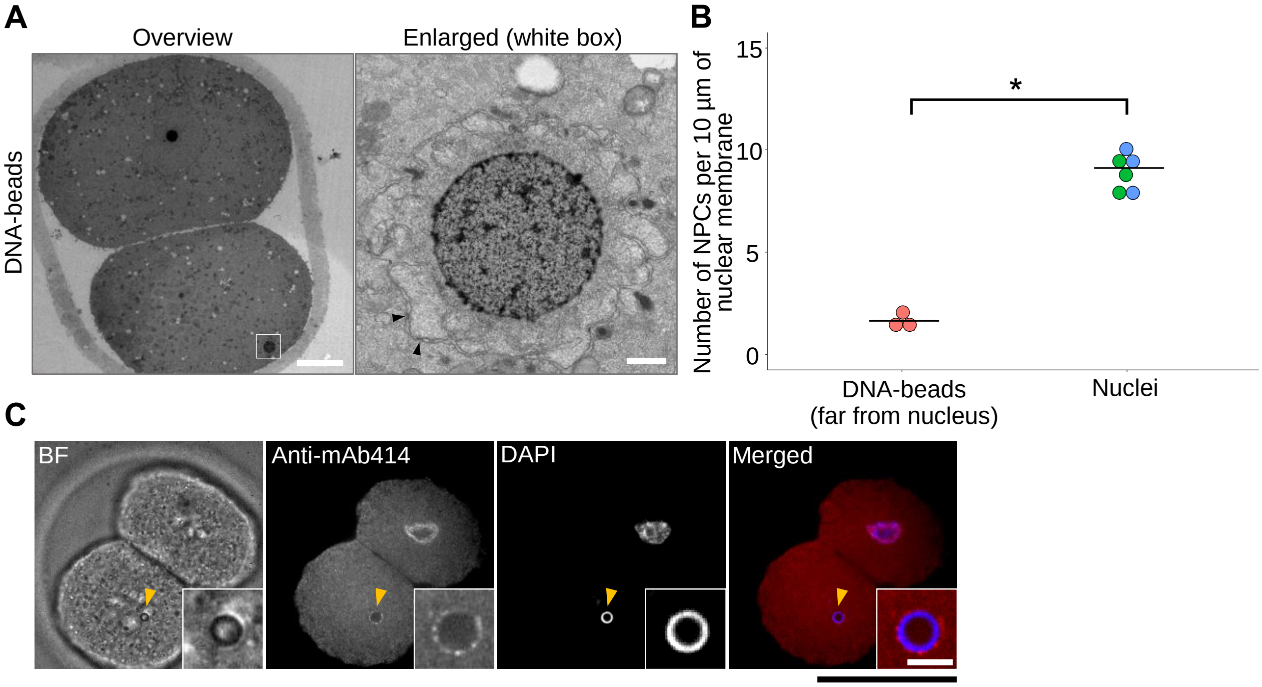

Fig. S1. Few nuclear pore-like structures were observed around DNA-beads far from the nucleus. (A) Electron microscopy images of DNA-beads located away from the nucleus. Scale bar = 10 µm (overview) and 0.5 µm (white box in overview). Arrowheads indicate the nuclear pore-like structures. (B) Comparison of the densities of pore-like structures on the membranes between DNA-beads and native nuclei. Red, green and blue dots show the numbers of pores on the DNA-beads, the native nuclei of embryos injected with DNA-beads, and the native nuclei of embryos injected with control-beads, respectively ($P = 0.02$ by Wilcoxon rank sum test; the data for native nuclei are the same as those shown in Fig. 4C). (C) Images of immunostaining using mAb414 to localize NPCs. For each, bright field (BF), anti-mAb414, DAPI and merged images are shown from left to right. Arrowheads indicate the position of beads. Scale bars = 50 µm (overview) and 5 µm (enlarged: lower right).

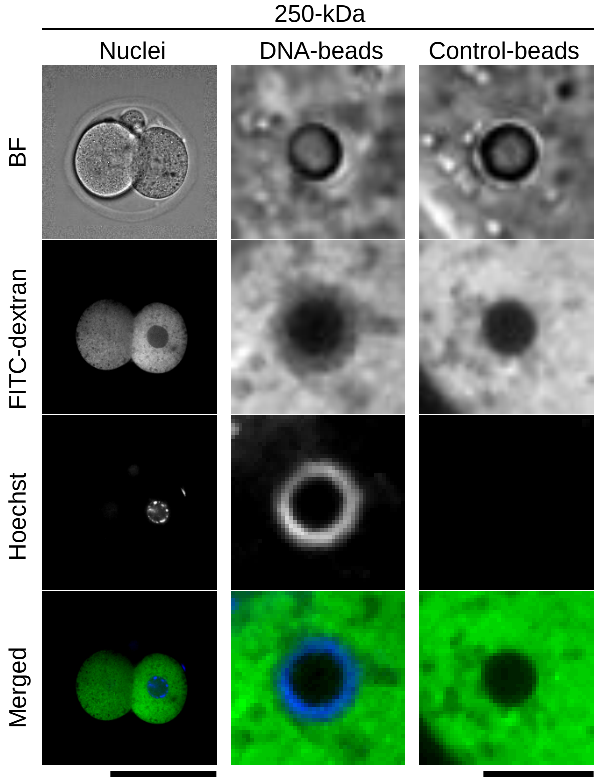

Fig. S2. Nuclear membrane-like structures covered the entire surface of DNA-beads. Representative images of the permeation of 250-kDa FITC-dextran in DNA-beads and control-beads. In each, bright field (BF), FITC-dextran, Hoechst33342 DNA staining and merged images are shown from top to bottom. Scale bars in the panels showing nuclei and beads are 50 μm and 5 μm, respectively.

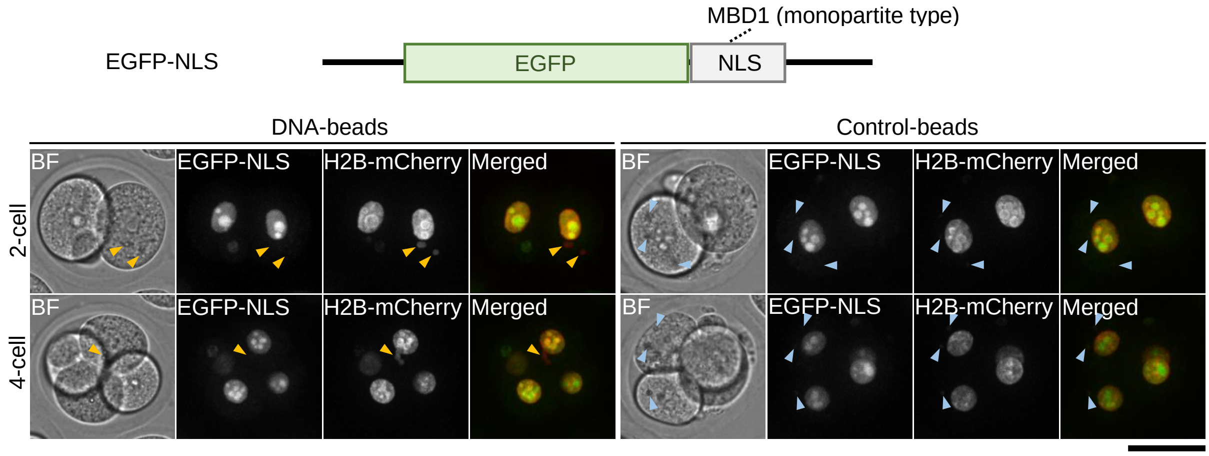

Fig. S3. The 'nuclei' around DNA-beads do not show nuclear transport of EGFP-NLS. The top drawing shows the structure of EGFP-NLS; this NLS is a monopartite NLS originating from methyl-CpG-binding domain protein 1 (MBD1) (*39*). Images show fluorescent images of 2- and 4-cell stage embryos expressing EGFP-NLS around DNA-beads and control-beads. In each, bright field (BF), EGFP-NLS, histone H2B-mCherry and merged images are shown from left to right. Upper and lower panels show the 2-cell stage and 4-cell stage embryos, respectively. Scale bar = 50 µm. The numbers of fertilised eggs used for each experiment are listed in Supplementary Table S3.

Supplementary Table S1

Development of Embryos Injected With Beads

	No. of beads introduced	No. (%) of surviving fertilized eggs	No. (%) of embryos developed for 24 hour			
			2-cell	4-cell	Morula	Blastocyst
DNA-beads	3	54/56 (96)	53/54 (98)	46/54 (85)	45/54 (83)	45/54 (83)
	6	48/54 (89)	46/48 (96)	37/48 (77)	29/48 (61)	30/48 (63)
	9	48/54 (89)	39/48 (81)	22/48 (46)	15/48 (31)	18/48 (38)
Control-beads	3	45/50 (90)	43/45 (96)	43/45 (96)	44/45 (98)	42/45 (93)
	6	45/47 (96)	41/45 (91)	40/45 (89)	43/45 (96)	40/45 (89)
	9	46/54 (85)	45/46 (98)	43/46 (93)	42/46 (91)	39/46 (85)
Not injected	-	-	37/37 (100)	37/37 (100)	37/37 (100)	37/37 (100)

Supplementary Table S2

Full-Term Development of Embryos Injected With Beads

	No. of beads introduced	No. of transferred Blastocysts	No. of recipients	No. (%) of embryos implanted	No. (%) of newborn mice
DNA-beads	3	86	5	47 (55)	31 (36)
Control-beads	3	108	6	62 (57)	43 (40)
Not injected	−	60	3	32 (53)	30 (50)

Supplementary Table S3

List of antibodies and mRNAs used in this experiment

Target Proteins	Probe	Source	Antibody dilution and mRNA concentration	Rate of positive-proteins on beads-injected embryos		
				Nuclei	DNA-beads	Control-beads
Histone H2A	Anti-Histone H2A antibody	MBL, D210-3	×100	31/31 (100%)	22/22 (100%)	0/9 (0%)
Histone H3	Anti-Histone H3 mAb	Monoclonal Antibody Laboratory Inc., MABI0301	×400	29/29 (100%)	18/18 (100%)	0/11 (0%)
Histone H4	Anti-Histone H4 mAb	Monoclonal Antibody Laboratory Inc., MABI0400	×400	4/4 (100%)	3/3 (100%)	0/1 (0%)
Histone H3 Ser10phospho	Alexa 488 labeled mouse-anti-Histone H3 Ser10phospho Fab fragment	Hayashi-Takanaka et al., *J. Cell Biol* , 2009	15.6 µg/mL	12/12 (100%)	12/12(100%)	0/12 (0%)
Autophagy	Anti-LC3 antibody	MBL, PM036	×500	0/37 (100%)	0/22 (0%)	0/15 (0%)
Inner nuclear membrane	Anti-LaminB1 antibody	Santa Cruz Biotechnology, sc-20682	×100	34/34 (100%)	0/24 (0%)	0/10 (0%)
NPC	Anti-mAb414 antibody	BioLegend, 902901	×500	40/40 (100%)	24/25 (96%)	0/15 (0%)
Ran	Anti-Ran antibody	BD Biosciences, BD610341,	×250	15/15 (100%)	0/10 (0%)	0/5 (0%)
Inner nuclear membrane	Anti-LEMD2 antibody	Atlas Antibodies, HPA017340	×100	27/27 (100%)	16/20 (80%)	0/7 (0%)
NPC	Anti-ELYS antibody	Abcam, ab14431	×100	16/16 (100%)	8/9 (89%)	0/7 (0%)
Histone H2B	H2B-mCherry mRNA	Ueda et al., *Stem Cell Rep.* , 2014	5 ng/µL	112/112 (100%)	78/78 (100%)	0/34 (0%)
Nucleosome	RCC1-EGFP mRNA	This study	5 ng/µL	29/29 (100%)	19/19 (100%)	0/10 (0%)
Inner nuclear membrane	EGFP-BAF mRNA	Haraguchi et al., *J. Cell Sci.* , 2008	5 ng/µL	24/24 (100%)	16/16 (100%)	0/8 (0%)
NLS	EGFP-NLS mRNA	Kobayashi et al., *Development* , 2016	5 ng/µL	45/45 (100%)	0/33 (0%)	0/12 (0%)
NLS	GST-NLS-EGFP mRNA	Haraguchi et al., *J. Cell Sci.* , 2000	5 ng/µL	33/33 (100%)	0/25 (0%)	0/8 (0%)
